# Supplementary material for: Poor Generalization by Current Deep Learning Models for Predicting Binding Affinities of Kinase Inhibitors
Source: bioRxiv. 2023 Sep 6:2023.09.04.556234. Preprint. [Version 1] doi: 10.1101/2023.09.04.556234 (PMC10508770; doi:10.1101/2023.09.04.556234)
Supplement: Supplement 1 [file NIHPP2023.09.04.556234v1-supplement-1.pdf]

bioRxiv preprint doi: https://doi.org/10.1101/2023.09.04.556234; this version posted September 6, 2023. The copyright holder for this preprint (which was not certified by peer review) is the author/funder, who has granted bioRxiv a license to display the preprint in perpetuity. It is made available under aCC-BY 4.0 International license.

Supplemental Information

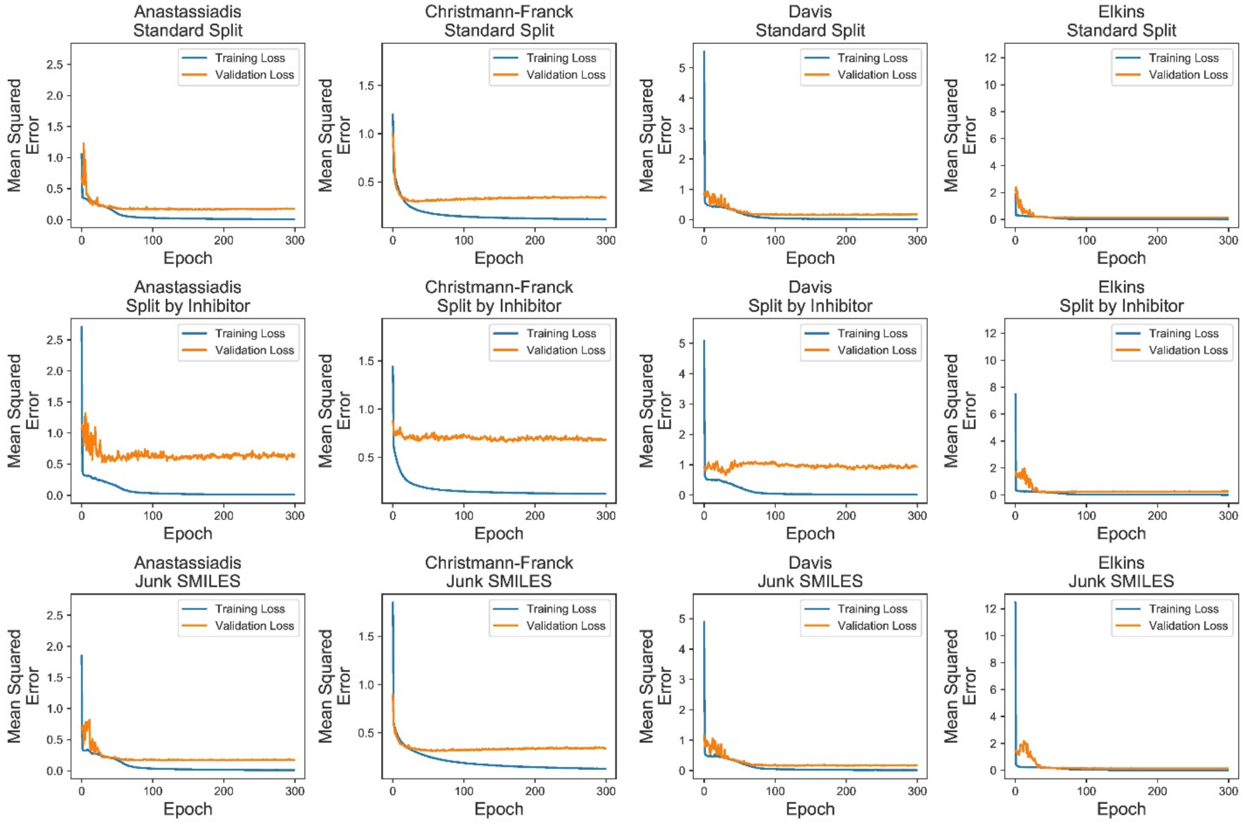

Figure S1: Training Set and Validation Set Loss Plots for Neural Network Model. We use the settings with lowest validation loss in the further evaluation of our model.

bioRxiv preprint doi: https://doi.org/10.1101/2023.09.04.556234; this version posted September 6, 2023. The copyright holder for this preprint (which was not certified by peer review) is the author/funder, who has granted bioRxiv a license to display the preprint in perpetuity. It is made available under aCC-BY 4.0 International license.

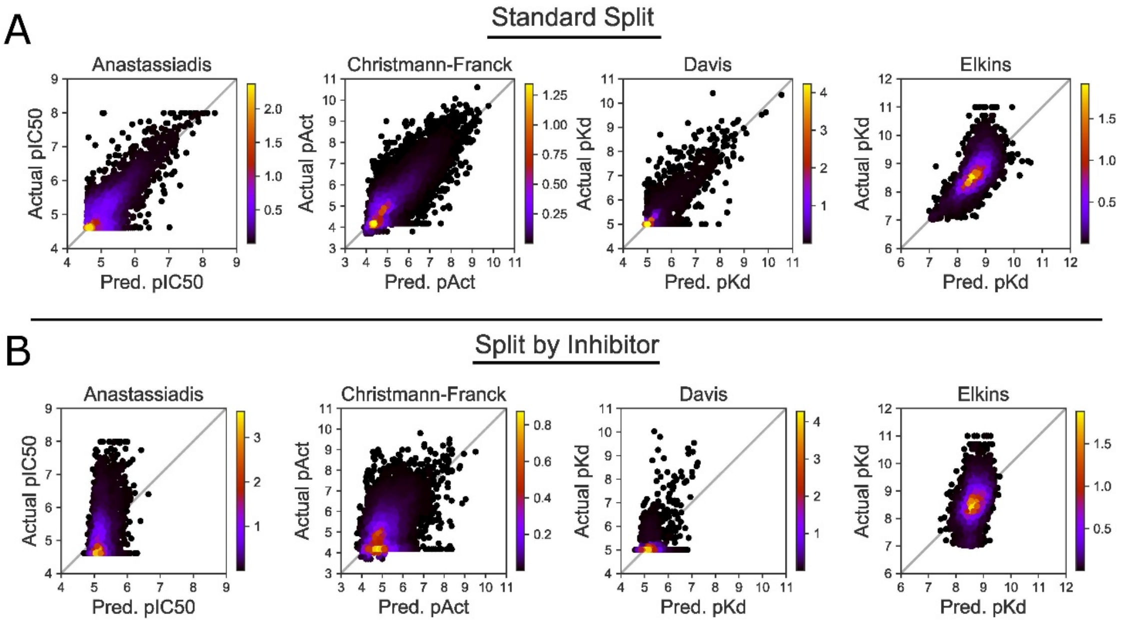

Figure S2: Using "Split by Inhibitor" diminishes model performance relative to "Standard Split". These scatterplots correspond to the same data shown in **Figure 3** as density plots.

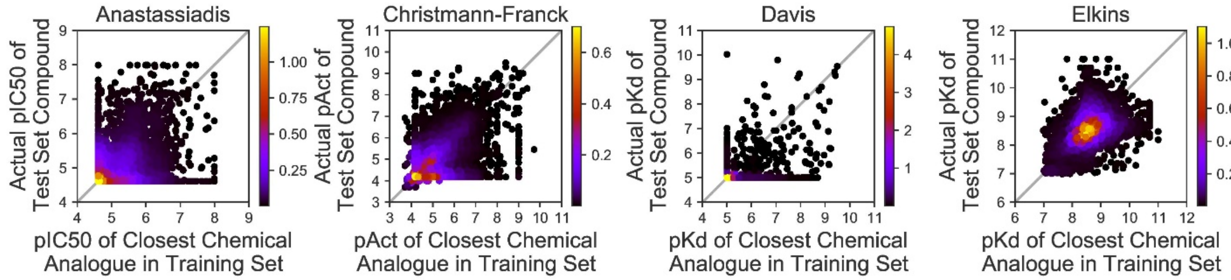

Figure S3: Using a tokenized inhibitor encoding does not diminish model performance. These scatterplots correspond to the same data shown in **Figure 4** as density plots.
